# Policy content and stakeholder network analysis for infant and young child feeding in India

**DOI:** 10.1186/s12889-017-4339-z

**Published:** 2017-06-13

**Authors:** Seema Puri, Sylvia Fernandez, Amrita Puranik, Deepika Anand, Abhay Gaidhane, Zahiruddin Quazi Syed, Archana Patel, Shahadat Uddin, Anne Marie Thow

**Affiliations:** 1 Department of Nutrition, Institute of Home Economics, University of Delhi, New Delhi, India; 2 National Institute of Nutrition, Hyderabad, India; 3 Lata Medical Research Foundation, Nagpur, India; 4 Community Medicine, Datta Meghe Institute of Medical Sciences, Wardha, India; 5 Center of Excellence, School of Epidemiology and Public Health, Datta Meghe Institute of Medical Science, Wardha, India; 6 Complex Systems Research Group, The University of Sydney, Sydney, Australia; 7 Menzies Centre for Health Policy, Sydney School of Public Health, The University of Sydney, Sydney, Australia

**Keywords:** Infant and young child feeding, Policy mapping, Stakeholder network analysis

## Abstract

**Background:**

Over the last decade, infant and young child feeding (IYCF) indicators in India have improved. However, poor IYCF practices are still apparent, associated with pervasive high rates of child under-nutrition. Interventions to improve IYCF need augmentation by appropriate policy support to consolidate gains. The aim of this study was to identify opportunities to strengthen and support IYCF policies through a policy content and stakeholder network analysis.

**Methods:**

IYCF policies and guidelines were systematically mapped and coded using predetermined themes. Six ‘net-map’ group interviews were conducted for stakeholder analysis with data analyzed using ORA (organizational risk analyzer, copyright Carley, Carnegie Mellon University) software. The study was carried out at a national level and in the states of Maharashtra and unified Andhra Pradesh.

**Results:**

Thirty relevant policy documents were identified. Support for IYCF was clearly apparent and was actioned within sectoral policies and strategic plans. We identified support for provision of information to mothers and caregivers in both sectoral and high-level/strategic policy documents. At a sectoral level, there was support for training health care workers and for enabling mothers to access IYCF. Opportunities to strengthen policy included expanding coverage and translating policy goals into implementation level documents.

At the national level, Ministry of Women and Child Development [MoWCD], Ministry of Health and Family Welfare [MoHFW] and the Prime Minister’s Nutrition Council [PMNC] were the most influential actors in providing technical support while MoHFW, MoWCD, and Bill Melinda Gates Foundation were the most influential actors in providing funding and were therefore influential stakeholders in shaping IYCF policies and programs.

**Conclusion:**

We identified a wide range of strengths in the IYCF policy environment in India and also opportunities for improvement. One key strength is the integration of IYCF policies into a range of agendas and guidelines related to health and child development service delivery at the national and state level. However, the lack of a specific national policy on IYCF means that there is no formal mechanism for review and monitoring implementation across sectors and jurisdictions. Another opportunity identified is the development of IYCF policy guidelines in emergencies and for tribal populations.

**Electronic supplementary material:**

The online version of this article (doi:10.1186/s12889-017-4339-z) contains supplementary material, which is available to authorized users.

## Background

Globally, over half of child deaths associated with under nutrition are attributed to inadequate breastfeeding and/or complementary feeding [1]. The period from birth to two years of age is recognized as the “critical window” for promotion of good growth, behavioural and cognitive development [2]. To accelerate progress on child survival there is heightened global interest in increasing rates of optimal infant and young child feeding (IYCF) practices, that include early initiation of breastfeeding, exclusive breastfeeding for the first six months of life, and beyond six months, timely and age-appropriate complementary feeding, with continued breastfeeding up to two years of age [1, 3]. WHO and UNICEF jointly adopted the Global Strategy for IYCF to focus on impact of feeding practices on survival, nutritional status and growth and development of infants and young children [4, 5]. Recognizing the essential role of nutrition in improving health outcomes, attention has been drawn to enhancing nutrition in mothers and children, as in the recent United National Sustainable Development Goals (SDG 2) [6, 7].

In India, IYCF practices have improved substantially over the past decade, but still remain sub-optimal [8–12]. In 2012, the prevalence of exclusive breastfeeding to age six months was 65%, up from 46% in 2005–2006, but with wide variation between states. Similarly, rates for early initiation of breast feeding in 2012 varied from 32 to 84% between states with an average of 58%, up from 40% in 2008 [DLHS-3] [13–15]. Introduction of complementary feeding along with continued breastfeeding in infants aged 6–9 months was 55.8%, up from 35% in NFHS 2 [16]. The critical challenges in India are to support early initiation of breast feeding, exclusive breast feeding, and optimum complementary feeding [15–17].

Effective interventions to promote optimum breastfeeding and complementary feeding practices, including counseling, food supplementation and food based comprehensive approaches, micronutrient interventions and general supportive strategies, are being established [8, 9]. However, for best outcomes at a national level, such interventions need to be supported by appropriate policies and guidelines. Key areas where policy can enhance IYCF interventions are through support to women at home and in the work place, consistent messages regarding IYCF, training of health care workers to deliver IYCF interventions, and ensuring that policies for promotion, protection and support for IYCF practices are consistently supported in implementation level documents [18].

The World Breastfeeding Trends Initiative [WBTi] report of 2015 [17] revealed that policies and programs in India that support IYCF practices showed insignificant progress on most indicators [17]. In fact, key indicators on national policy and coordination, Baby Friendly Hospital Initiative [BFHI] and implementation of the International Code showed a decline in performance.

In India while policies are generally formulated at the national level, the actual implementation of these policies lies with the State governments. The WBTi report also reiterated that there was lack of effective coordination mechanisms at central or state government levels, and little information regarding the content of IYCF policies or the role and influence of stakeholders and institutions in national policy making [17]. An understanding of the existing policy landscape, the content and the contribution of stakeholders is therefore imperative to identify opportunities for not only effective policy formulation but also its implementation.

This study builds on previous research [19] and provides a more detailed review of the content of existing IYCF policies and guidelines in India at both national and state levels, including implementation documents, focusing on how policies enable caregivers to engage with best-practice interventions.

Although various stakeholders influence policy and program guidelines for IYCF, either directly or indirectly through technical or funding support, little is known about them [20]. Analysis of the Adoption of the Global Strategy for IYCF found a systematic lack of attention by policy makers and program planners to enhancing optimal breastfeeding practices [4, 19]. Hence, this research aims to analyze the influence of various stakeholders/actors in shaping IYCF policies and program guidelines. Finally, based on both the content analysis and stakeholder mapping, we identified opportunities to strengthen policies to support appropriate IYCF.

## Method

This research was part of a larger study undertaken by South Asia Infant Feeding Research Network (SAIFRN), designed to review policy documents and map current policies that support IYCF in Bangladesh, India, Nepal, Pakistan and Sri Lanka. Policy documents were reviewed in relation to both breastfeeding and complementary feeding. We analyzed how policy decisions are implemented comparing various approaches and implementation data with outcomes using the Walt and Gilson policy analysis framework [21]. We had a focus on analysis of content (through the policy matrix), of actors (through stakeholder mapping) and of context (both of the above, plus additional contextual information) [14].

The study was carried out at the national level in New Delhi and state level in Maharashtra and Unified Andhra Pradesh. Ethics approval was obtained from the Institute of Home Economics, Delhi, India; Datta Meghe Institute of Medical Sciences, Nagpur, India, and National Institute of Nutrition, Hyderabad, India. The study was conducted in two phases, content analysis of IYCF policy and program guidelines followed by stakeholder analysis.

### Data source and data collection

Both content and actors are key in understanding the policy landscape and identifying opportunities for policy change.

### Content analysis

We used a mapping approach to identify relevant policy document types and sectors that could incorporate support for IYCF to guide comparable policy content data collection ranging from whole-of-government strategic policy documents to implementation guidelines relevant to IYCF.

We searched websites of relevant government departments, institutes and ministries for policy documents and guidelines. Search terms used were: Infant and Young Child Feeding, Breastfeeding, Breast feeding, Exclusive breastfeeding, Complementary feeding, IYCF counseling, Maternity benefit schemes, Policies, Guidelines, IYCF trainings, National Health Policy, National Nutrition Policy, and National Population Policy. In addition to a web search, investigators contacted key personnel in government and non-government agencies relevant to IYCF and health service provision to request relevant documents. For each document identified as relevant, we recorded source, purpose, relevant dates, and support for IYCF.

### Stakeholder analysis

For stakeholder analysis, Net-Map was used, which is a group interview tool to examine linkages, influences and goals of actors in policy [22]. This was undertaken to identify actors influential in shaping IYCF decisions, the links with each other in regard to technical and funding support, and their position and how strongly they influenced policy and program decisions. Eight Net-Map group discussions were conducted in Delhi, representing the national level, and two each in Maharashtra and Unified AP.

We chose the Net-Map tool because it is an interview-based mapping tool that helps people understand, visualise, discuss and improve situations in which many different actors with variable influence levels can influence outcomes. We mapped the most influential actors in the IYCF policy landscape in regards to how these actors are linked to each other for technical and funding support activities. In order to derive the links, we defined the technical and funding links. First, we discussed with the group/participants regarding the actors that may influence the IYCF policy landscape at the National and the State levels. Subsequently, we derived a list of probable actors and placed each actor separately into the blank Net-Map Sheet. We then discussed with the group regarding how these actors are linked with each other for technical and funding support, including the direction of link (who to whom) in order to draw links between actors on the Net-Map sheet with different colour for funding and technical support. Next, to visualize the level of influence of each actor, we placed a stack of coins over each actor after through discussion with the group. By visually capturing these arrangements, the Net-Map tool assists policy makers and program managers (participants) in understanding how actor networks influence policy and programs to promote, protect and support IYCF.

### Recruitment of participants for net-map group discussions

A list of potential participants, groups of people or organizations thought to be knowledgeable or experienced in IYCF policy and programs were identified. The research team contacted those identified as knowledgeable and lists of potential participants for group discussion were generated by snowballing. The final list of 96 potential participants was reviewed to ensure participants were representative of sectors related to IYCF policies and programs. Participants were contacted through email or telephone and sent a formal letter of invitation.

An actor who had an effect on allocation of resources was defined as “an actor influential in ‘shaping’ decisions”, i.e. shaping policy or program selection or design. Technical support or linkages were defined as a formal exchange of technical support [training, technical guides, equipment, staff/consultant [preselected], information, research evidence, routine data or field experience/case studies. Funding links were defined as exchange of funds or in-kind support related to IYCF [funding or supplies for programs or staff employed by the recipient, or budget provision]. This excluded provision/funding of preselected staff, which would fall in the technical link. We considered actors’ influence in the past 5 years. This meant, but was not limited to: formal supervision, funding, technical information, advice, advocacy and pressure, but might go beyond these links, e.g. influence because one is respected.

### Data analysis

#### Content analysis

We developed a matrix for assessing policy support based on four themes focused on caregiver engagement with IYCF interventions:

1. High level policies providing strategic/general support
2. Provision of standardized and correct information to mothers and care-takers
3. Training of health workers to counsel mothers
4. Enabling mothers to engage with health care workers and best practice interventions (e.g. maternity leave).

We analyzed the data using a narrative synthesis approach focusing on the presence of IYCF in policy documents, how comprehensive the approach was (e.g. different sectors, avenues), and the translation of high-level policy statements into implementation-relevant documents. Narrative/thematic synthesis using an integrated critical perspective [23] was used to describe the content related to IYCF, within the umbrella of nutrition more broadly, for each policy document identified. This protocol was not closed during data collection and themes were built up inductively over the research process.

### Stakeholder analysis

Net-Maps were analyzed quantitatively using ORA (Organizational Risk Analyzer, copyright Carley, Carnegie Mellon University) software using Social Network Analysis [SNA] to map and measure relationships among actors and explore relative positions. The four basic centrality measures are:

- **In-degree centrality** which quantifies the tendency of an actor to receive input from the other network actors [24] and is a measure of receptivity or popularity
- **Out-degree centrality** which quantifies the tendency of an actor to provide input to other network actors and is a measure of expansiveness or activity in a network [24]
- **Betweenness centrality** that represents the capacity of an actor to control the flow of information between any pair of member actors [24]. The underlying assumption is that “actors in the middle” have more “interpersonal influence” on others.
- **Closeness centrality** that represents the reachability and level of connectivity of an actor from the other actors [24] that is a path linking them. If an actor is close to all other network actors, then – i] the other actors can reach that actor with minimum effort; and/or ii] that actor will need less effort to communicate with the other actors.

## Results and discussion

### Content analysis

A total 33 documents were retrieved from the governments of India, Maharashtra and Unified Andhra Pradesh. Thirty relevant policy documents, plan of actions and guidelines were included in the analyses; three policy documents that were related to nutrition and had no reference to IYCF were excluded. Two of the relevant documents were five-year strategic plans of the Government of India; three were legislation related to maternity benefits, one each was on the Infant Milk Substitute (IMS) Act and the National Code for Promotion and Protection of Breast Feeding. Six were national policy documents; six were national guidelines and six were national programs related to IYCF. One document was specific to Maharashtra and 4 specific to unified Andhra Pradesh (Additional file 1: Table S1). We identified support for IYCF in all of the domains identified, which are described below.

### General/strategic support for infant and young child feeding

General support ranged from strategic whole-of-government support for IYCF to the strengthening of grass root level institutions providing care to mothers and young children, enhanced budgetary support for strengthening infrastructure, manpower and training within the health system, and the promotion of IYCF in institutions and home deliveries. At the state level, IYCF was explicit in health and nutrition programs in unified Andhra Pradesh and Maharashtra.

There was clear support for IYCF in the Twelfth Five Year Plan [2012–2017] by the Planning Commission Government of India [2012], which recommended strengthening National and State Coordination Mechanisms and Capacity for promoting IYCF, for example by organizing “Village Health and Nutrition Days”, and improving the implementation and monitoring of the Infant Milk Substitute Act [25]. In line with this, the Five Year Strategic Plan [2011–2016] of the Ministry of Women and Child Development [MoWCD] [26] included specific provisions for strengthening the Integrated Child Development Services Scheme [ICDS] and enhancing budgetary support for promoting exclusive breastfeeding and complementary feeding. ICDS is the flagship children’s program of the Indian government and has been restructured to focus on children under 3 years, emphasize IYCF and target the first 1000 days [26, 27]. This strategic direction is actioned within the National Policy for Children [28, 29], which builds on the National Plan of Action for Children [Ministry of Human Resource Development, 2004] [30] to guide and inform all laws, policies, plans and programs affecting children, including IYCF. One objective is to promote breast-feeding to ensure optimal early nutrition. However, the IYCF recommendations fail to receive adequate priority within the implementation documents related to child care, which focus on issues such as immunization, prevention and treatment of diarrhoea and respiratory infection. Under the Health System Strengthening Initiatives in the National Population Policy, efforts for IYCF include the provision of special benefits to women and children through the public health system and promotion of reproductive and child health through counseling and mobile clinics [31].

General strategic policy support is followed up in the two states we assessed. In Maharashtra State, Rajmata Jijau Health and Nutrition Mission [32] was launched by MoWCD in 2005 to reduced malnutrition in 0–3 year-old children. In unified Andhra Pradesh, Maarpu Programme, an Interdepartmental Coordination for Effective Convergence recommends support for IYCF through special care and supervised feeding of malnourished children. This includes provision of a locally developed weaning food [“balamurtham], introduced under ICDS to provide improved supplementary nutrition to children aged 7 months to 3 years [33].

High level support for IYCF is also provided in the Indian National Code for Protection and Promotion of Breast Feeding [34] and IMS Act [35], which have codes for planning, provision, design and dissemination of information, correct marketing practices, control on manufacturers of breast milk substitutes, and which provide legal frameworks for regulatory approaches to promote breast feeding. The IMS Act contains all provisions of the ‘International Code of Marketing of Breast Milk Substitutes’ and its scope expands to all foods for children up to the age of 2 years. The IMS Act also includes relevant resolutions of the World Health Assembly [35].

### Provision of correct information to mothers/caregivers

Provision of correct information on IYCF to mothers/caregivers through counseling at hospital, health centres and community outreach through Village Health and Nutrition Days [VHND] is emphasized by 12th Five Year Plan of Government of India [25]; Indian National Code for Promotion and Protection of Breast-feeding [34]; National Population Policy [31]; Guidelines for Enhancing Optimal IYCF – 2013 [36]; National Guidelines for IYCF [2003 revised 2006] [37]; Operational Guidelines on Facility Based Management of Children with Acute Severe Malnutrition [38]; Operational Guidelines of Nutrition Rehabilitation Centres [39]; and Navjaat Shishu Suraksha Karyakam [NSSK] of the Government of India [40] [Table 1].

**Table 1:** Total actors in the technical and funding landscape at national and state level

	Total actors-technical	Total actors-funding
National (India)	87	44
Maharashtra State	63	32
United AP State	44	33

The 12th Five Year Plan specifically recommends a ‘Nutritional and Care Counseling’ service through ICDS. This represents a significant change within the ICDS program guidelines, with more focus on counseling in addition to their existing focus on providing correct information to caregivers and nutritional supplementation to pregnant and lactating women, and children. The National Action Plan provides a broader policy framework and is more of a guide for the development of implementation documents. At the implementation level, the 12th Plan advises setting up ‘Nutrition and Breastfeeding Support’ Centres in the health sector, with skilled counselors initially in all district hospitals, then in Community Health Centres and subsequently in Primary Health Centres in a phased manner [25]. The recent initiative regarding restructuring of ICDS, proposed a nutritional counselor and an additional Anganwadi worker in selected districts initially, and to be phased into the remaining districts.

Specific IYCF information will be provided to mothers, such as timely initiation of breastfeeding, exclusive breastfeeding and complementary feeding as stated in the Dietary Guidelines for Indians [41], National Guidelines on Infant and Young Child Feeding [37], Operational Guidelines for Facility Based Management of Children with Severe Acute Malnutrition [38], Operational Guidelines for Nutrition Rehabilitation Centres [39], Neonatal and Childhood Illness Guidelines [42]. These documents provide consistent information that supports implementation of the National Plan of Action on Children [30], which specifically identifies the need for feeding colostrum in home and hospital delivery; early initiation of breast-feeding; exclusive breastfeeding for children from birth to six months with continued breastfeeding up to two years or beyond; and promotion of complementary feeding after six months. Provision of correct information to mothers is also supported by the IMS Act [35] and restricts provision of information related to infant milk substitutes by companies. These guidelines were cross-referenced with strategies in policy documents from the Ministry of Women and Child Development [ICDS] and Ministry of Health and Family Welfare [NRHM], which specify that Anganwadi workers [AWW] and health care providers at all level shared responsibilities for providing information and counseling on IYCF [43].

At state level in Maharashtra, the Rajmata Jijau Health and State Nutrition Mission, a technical, advisory and training body recommends health staff and community workers to give demonstrations regarding child nutrition and feeding to mothers at Village Child Development Centres (VCDC) or Nutritional Rehabilitation Centres located at block or district health facilities [32].

### Training of health workers for infant and young child feeding

There is policy support for the training of service providers, from doctors to peripheral health and community workers, as separate or stand-alone IYCF training guidelines or by integrating IYCF components into other training curricula. Training, which is mainly delivered by the government jointly with the Breast Feeding Promotion Network of India (BPNI) and UNICEF [18], uses a “train the trainer” approach to health workers, who counsel in the community, and to doctors and nurses in hospitals [18].

The 12th Plan document [25], National Policy for Children [28] and the National Health Policy [44] support promotion of optimal IYCF practices though counseling. Frontline workers are required to obtain competencies for IYCF counseling and deliver counseling through Anganwadi Centres. The policies provide additional support in terms of human resources, Information Technology and Management Information Support, as well as capacity building of Anganwadi Workers for promoting IYCF.

The 12th Five Year Plan recommended strengthening the curriculum of IYCF pre-service training for doctors and nurses and suggested the creation of “Centres of Excellence” in IYCF, forming hubs in order to strengthen institutions [25]. The Guidelines [2013] [36] recommended IYCF as a key component in pre-service and in-service training of Integrated Management of Neonatal and Childhood Illness [IMNCI] and in facility based management of IMNCI for Doctors and Nurses, Skilled Birth Attendance Training, Training of Accredited Social Health Activist (ASHA training module 6 and 7, which addresses IYCF [45, 46]). Training, with an emphasis on building counseling skills, is recommended in Guidelines for Community Health Workers and Nutritional Counselors [37] and National Plan of Action for Children [30]. The Operational Guidelines for Nutritional Rehabilitation Centres [2011] [39] also specifically mention training of health workers in behavior change communication for nutritional counseling and IYCF. The ICDS program recommends a common core joint training module for ICDS and RCH, which will include the National Guidelines on IYCF [37]. The document also recommended incorporating training packages, especially, for IYCF counseling in medical and nursing curricula. National AIDS Control Organization supports the specific training of health workers to counsel mothers with HIV infections regarding pregnancy and breastfeeding [47].

### Enabling mothers/caregivers to engage with the best care practice interventions

Caregivers are supported to engage with best practice interventions through the provision of accessible health services, promotion of institutional delivery with IYCF support, and initiatives to improve work-based support for breastfeeding and maternity leave.

IYCF counseling centres in health facilities, as recommended by 12th Five Year Plan [25], and counseling services through mobile clinics, as recommended under National Population Policy [48], enable mothers and caregivers to engage with best practice interventions. The National Population Policy and Janani Suraksha Yojana under the National Rural Health Mission promote institutional deliveries, which are a crucial opportunity for health workers to support women for early initiation of breast feeding and exclusive breastfeeding [49]. The Baby Friendly Hospital Initiative [BFHI] [50], a global initiative launched in 1991 by WHO and UNICEF to improve maternity services, also promotes early initiation of breast feeding and exclusive breast feeding for all births in hospital/facility during pregnancy and after delivery. The BFHI have a written breastfeeding policy that is routinely communicated to all health care staff [50]; they advocate for no artificial teats or pacifiers to be used on breastfeeding infants and they foster the establishment of breastfeeding support groups, referring mothers on discharge from the hospital or clinic.

Working women are supported to access IYCF through legislation supporting maternity leave and benefits [51]. The Maternity Benefit Act of Ministry of Labour & Employment in 1961 (amended 2017), Central Civil Services Leave Rules; 1972 [52], and Employees State Insurance Act 1948 [53] provides for maternity leave for women, in certain establishments for six months, and for maternity and other benefits. This Act is implemented in the government sector, but more effort is needed to ensure maternal protection in private or non-formal sectors. Currently the act does not support women in informal employment or women working in unorganized sectors such as farm workers, household staff etc. The issue of maternity protection is critical for the success of breastfeeding [53]. The National Food Security Act [NFSA] [54] also provides assurance of maternity protection for six months and covers women employed in the government sectors and in some non-government but organized sectors. In Maharashtra state, the Rajmata Jijau Nutrition Mission has, as a best practice intervention, established ‘Hirkani-kaksh’ [feeding/baby care rooms] at work places and public bus stops and Milk Banks for employed women [32].

Caregivers are also supported to engage with best practice interventions through provisions in the NFSA to increase food security for families with children, and to institute much-needed reforms for ICDS program strengthening and restructuring to promote and support optimum IYCF practices through Anganwadi cum Nutrition Counselors [54]. However, while the National Health Policy recommends providing special benefits to women and children through the public health system, it does not identify specific measures that directly promote and support IYCF. The National Population Policy 2000 [31] mentions the promotion of reproductive and child health through counseling and mobile clinics and recommends that the Baby Friendly Hospital Initiatives [50] should be extended to all hospitals.

### Supporting IYCF in specific situations

With regard to IYCF in special situations, the National AIDS Control Program published specific guidelines in 2005 for breastfeeding women infected with HIV and these have been revised in line with international recommendations [41]. The current Navsanjavani Yogana for Tribal areas in Maharashtra state includes some components on IYCF [40], but we did not identify other policy documents with reference to IYCF in tribal areas. However, in emergencies India follows the WHO guidelines for IYCF in emergencies [55].

### Opportunities to strengthen the policy landscape

Content analysis of IYCF policy documents and guidelines revealed that the overall policy environment in India seems conducive to the protection and promotion of optimal IYCF practices. Whole of government support was evident through restructuring the ICDS in the national development plan, and there was significant support within the health sector and the women and development sector. Support for provision of correct information to mothers is consistent across policy documents. Implementation was the responsibility of states and, within our two case study states, we identified policy documents supporting IYCF implementation in line with national guidelines.

However, we also identified opportunities to strengthen support and protect IYCF at a national and state level. The government has developed most IYCF strategies as guidelines with a corresponding plan of action, but these guidelines lack policy status. Giving policy status to them would ensure stringent regulatory provisions for effective implementation of IYCF recommendations. The current Program Implementation Plan of Health Mission at states may ensure an allocated budget for the promotion, support and protection of IYCF practices.

A further opportunity is to improve counseling support. The Operational Guidelines for IYCF [2013] [37] have made a provision for counseling services and capacity development but how counseling should be delivered and the resourcing, roles and responsibilities of staff at various levels requires more clarity. The Guidelines for Enhancing Optimal Infant and Young Child Feeding Practices, 2013 [36] recommend IYCF services are integrated within various levels of health care delivery; however, a guideline for IYCF community level actions is needed. Strengthening IYCF activities within communities needs to be a high priority action for promoting optimum IYCF practices. The guidelines should focus on initiatives for capacity building in counseling, and strengthening community level counseling in addition to health system level initiatives to promote and protect optimum IYCF practices. The National Policy for Children [28] and National Plan of Action for Children [30] could be strengthened by inclusion of IYCF as critical to child survival.

Our analysis also indicated that India needs more clear plan and policy guidelines regarding IYCF in emergencies and other specific situations. In particular, considering the different challenges and socio cultural context, separate guidelines for promoting IYCF in tribal areas may be useful. Legislation related to IYCF practices would be stronger with specific mentions about the mechanisms for monitoring and enforcement. More clarity would be required on a framework for easier and faster reporting of violations as well as capacity building and skilled training of officials involved in monitoring, and sensitization of judiciary and media.

### Stakeholder analysis

#### Overview

Overall 96 actors influential in shaping IYCF policies and program guidelines were identified across the study sites with a high degree of consistency between national and state regions. Most actors were from the Government sector (60%), followed by Development partners/International Agencies (20%), NGO/Civil Society (10%) and Research/Academic (10%) (Table 1).

In Net-Map diagrams, an actor is indicated by a node, links between actors are ties and directions of links are marked by arrows. The influence of actor is indicated by node size (larger nodes have higher relative influence). This study used four network measures – in-degree captures the tendency of an actor to receive input from other network actors; out-degree quantifies the tendency of an actor to provide support to other network actors; closeness centrality of an actor indicates its reachability by other network actors for any collaborative action; and betweenness centrality represents the importance of an actor in terms of its influence in the indirect communication (or collaboration) of any other pair of actors in the network.

#### National level

**Fig. 1:**
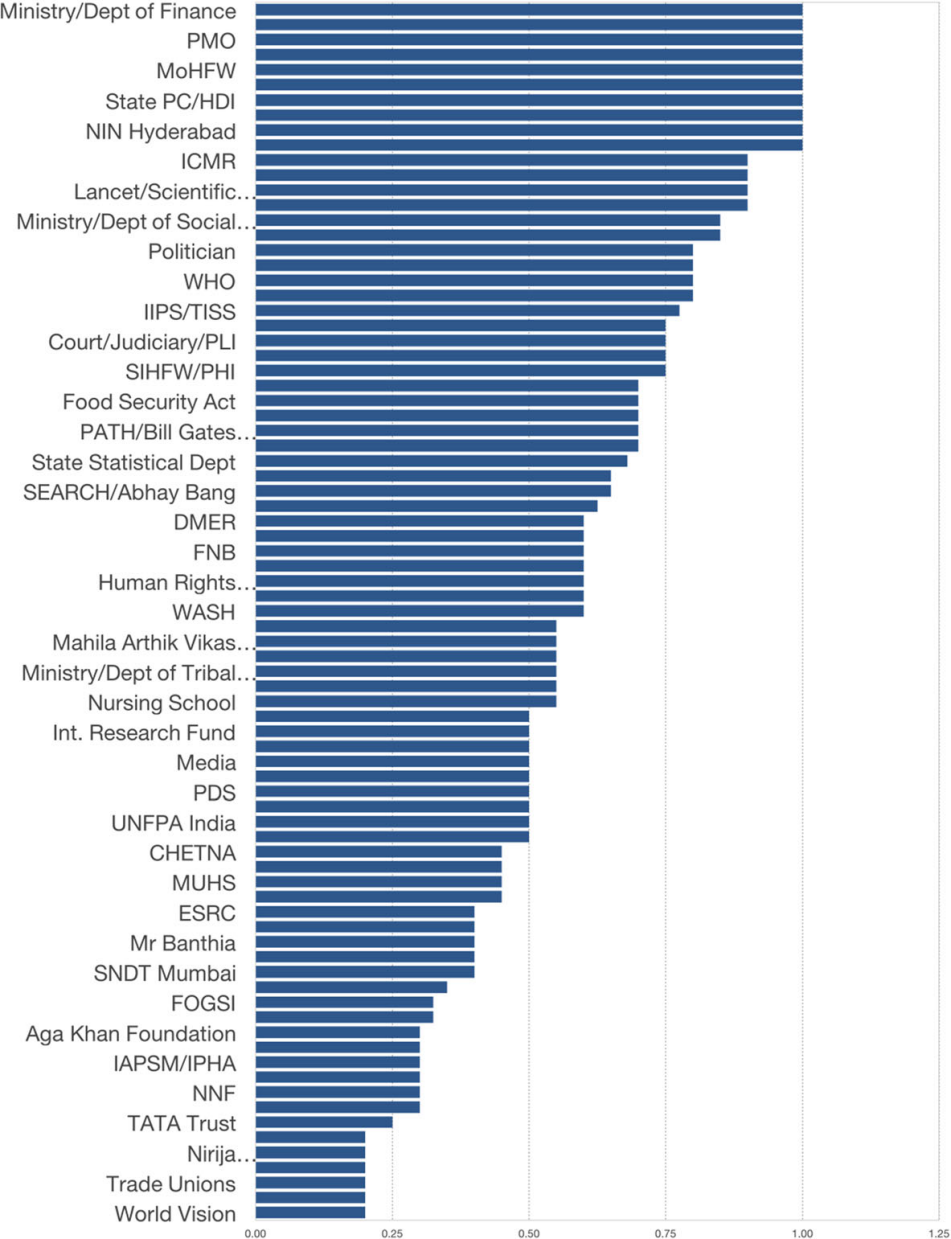
Relative influence of actors

**Fig. 2:**
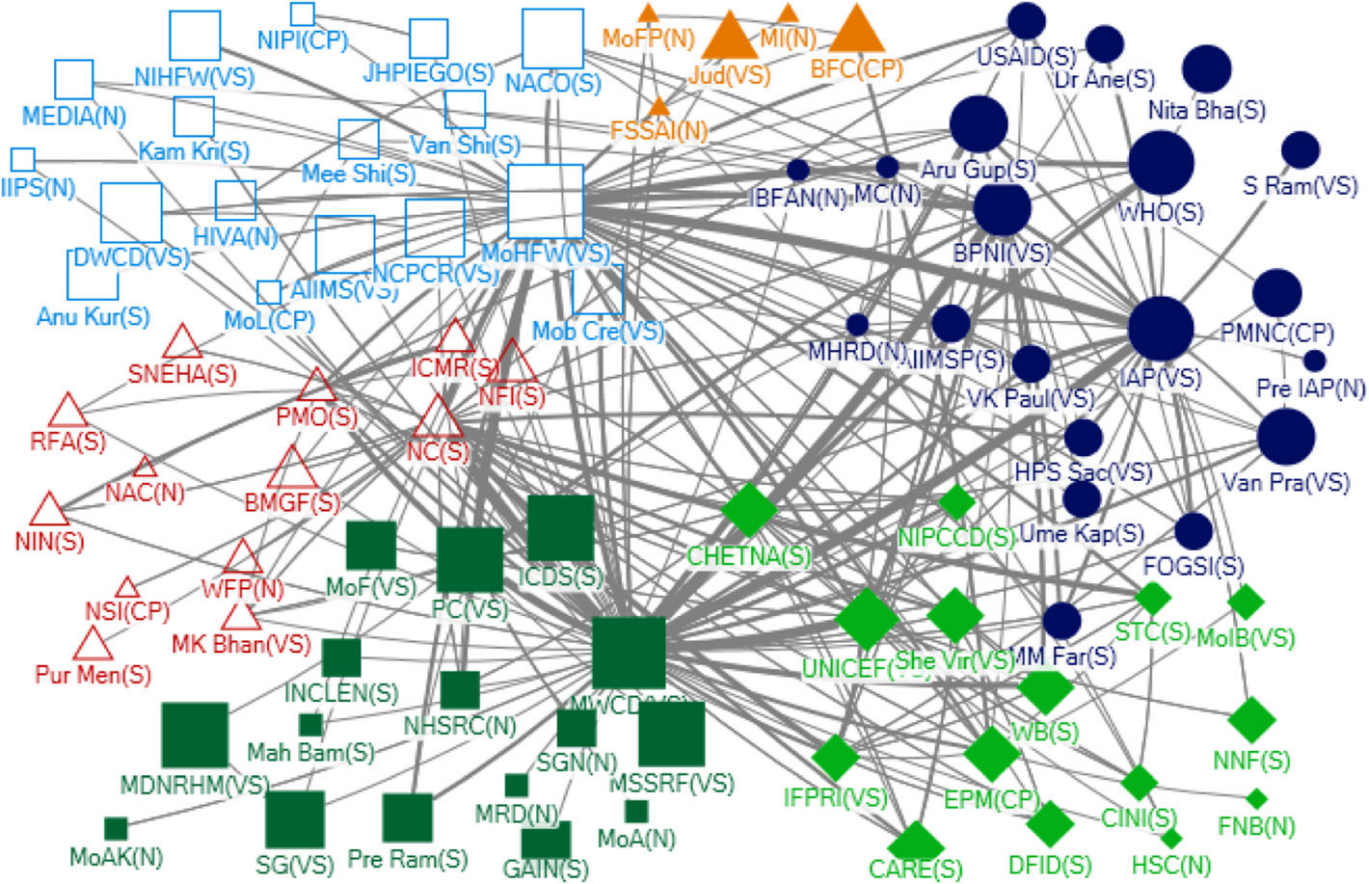
Community Network for Technical Support at National Level

The Ministry of Women and Child Development (MoWCD), Ministry of Health and Family Welfare (MoHFW), and Prime Ministers Nutrition Council (PM NC) had a great relative influence (Fig. 1) and were the most influential actors in provision of technical support (Fig. 2). MoWCD, MoHFW, Planning Commission, NIPCCD, NIN and ICMR play a major role in shaping the policy program of IYCF. These actors all received (in-degree) and provided (out-degree) substantial technical support, were accessible to a wide range of actors (closeness) and by their position in the network exerted a high degree of control over the flow of information between actors (betweenness) (Table 2). They were also ranked in the top 10 in relative influence (Fig. 1). Participants commented that ministries do not have a role in implementation of the acts and guidelines but are the law builders. One respondent stated

**Table 2:** Top five actors in the technical and funding network at national and state level

Netmap measures	Technical			Funding		
	National	Maharashtra	Unified AP	National	Maharashtra	Unified AP
In Degree Centrality	MoWCD (47)	MoHFW (39)	MoWCD (12)	BPNI (6)	MoHFW (10)	NIN (4)
	MoHFW (44)	MoWCD (33)	MoHFW (5)	MoHFW (6)	MoWCD (7)	Home science College (3)
	PM’s NC (26)	Public Health Dept (28)	AP Foods (2)	MoWCD (4)	NRHM (4)	MoWCD (3)
	BPNI (24)	NRHM (11)	Food and Nutrition Bureau (1)	PHFI (4)	Public Health Dept (4)	Corporates (2)
	IAP (17)	Rajmata Jijau (10)	Govt Hospitals (1)	NACO (4)	Medical Collages (4)	BPNI (2)
Out Degree Centrality	MoWCD (39)	BPNI (12)	NIN (6)	MoHFW (10)	MoFinance (10)	MoWCD (6)
	MoHFW (32)	UNICEF (11)	BPNI (4)	MoWCD (7)	Planning commission (8)	Science department (5)
	PM’s NC (25)	IAP (8)	Food and Nutrition Bureau (3)	BMGF (7)	ICMR (6)	UNICEF (4)
	IAP (20)	MoWCD (7)	ICMR (2)	ICMR (6)	World Bank (5)	IAP (1)
	BPNI (17)	ICMR (7)	UNICEF (2)	UNICEF (5)	UNICEF (5)	ICMR (1)
Betweenness	MoWCD (2908.105)	MoHFW (1625.226)	MoWCD (183.333)	MoHFW (993.704)	MoHFW (280.262)	MoWCD (75.333)
	MoHFW (2040.995)	MoWCD (968.108)	BPNI (70.000)	MoWCD (369.742)	Planning Commission (184.769)	Science department (28.333)
	PM’s NC (911.502)	Public Health Dept. (477.283)	NIN (30.667)	ICMR (362)	ICMR (144.252)	NIN (9.666)
	Planning Commission (658.406)	Nutrition Bureau (314.969)	MoHFW (19.167)	BMGF (289.428)	Medical College (118.75)	UNICEF (3.333)
	IAP (599.199)	UNICEF (200.339)	Food and Nutrition Bureau (9.667)	MoF (258)	MoWCD (113.145)	Home Science College (1.333)
Closeness	MoWCD (0.009)	MoHFW (0.012)	MoWCD (0.045)	MoHFW (0.013)	CSR (0.031)	NRHM (1)
	MoHFW (0.008)	MoWCD (0.011)	NIN (0.036)	BMGF (0.010)	MoHFW (0.20)	MEPMA (1)
	PM’s NC (0.007)	Public Health Dept (0.010)	BPNI (0.034)	UNICEF (0.010)	Planning Commission (0.019)	MoWCD (0.076)
	BPNI (0.007)	ICMR (0.009)	Food and Nutrition Bureau (0.031)	PHFI (0.01)	ICMR (0.018)	Science Department (0.058)
	Planning commission (0.006)	BPNI (0.009)	UNICEF (0.029)	MoF (0.01)	MoWCD (0.018)	NIN (0.055)

*“…ministry doesn’t implement. See you have the act but the implementation of the act is not monitored by the law ministry. It’s the activists, it’s like IAP or BPNI. These are the bodies. So, act itself would be like: a dowry prohibition act.”*

There were also two non-government actors identified as influential with respect to technical support. The Indian Association of Paediatrics (IAP), a professional association, and the Breastfeeding Promotion Network of India (BPNI) both received and provided technical support (Table 2). IAP was also ranked in the top 5 as being strategically positioned in the network (betweenness) and BPNI as reachable by a large number of actors (closeness). In net map discussion, it was evident that IAP provides technical guidelines but has a limited role in influencing policies.

There were six different communities in the technical support network at national level (Fig. 2). Two of the largest communities were centered on the MoHFW and MoWCD/ICDS respectively, with a third large community including WHO, BPNI and IAP. Actors with a very high level of influence tended to be well connected across other communities.

The MoHFW and MoWCD were the primary influencers with respect to funding. They provided funding (out-degree), received funding from other donors (in-degree), and exerted a high degree of control over the flow of funds between actors by their position in the network (betweenness) (Table 2). In addition, the MoHFW was accessible to a wide range of other actors (closeness). The Indian Council of Medical Research (ICMR), Bill and Melinda Gates Foundation and UNICEF were the most influential donors (out-degree; Table 2). Other agencies receiving (but not providing) funding (in-degree) were the Public Health Foundation of India (PHFI), a Public Private Partnership Initiative, which was accessible to a wide range of other actors (ranking highly for closeness), and the National AIDS Control Organisation (NACO) (Table 2).

The Ministry of Finance was identified as highly influential (Fig. 1) and within the funding network ranked in the top five with respect to being accessible to a wide range of actors (closeness) and exerting a high degree of control over the flow of funds between actors by their position (betweenness) (Table 2). The Planning Commission was a highly influential actor but was not well connected within the network.

**Fig. 3:**
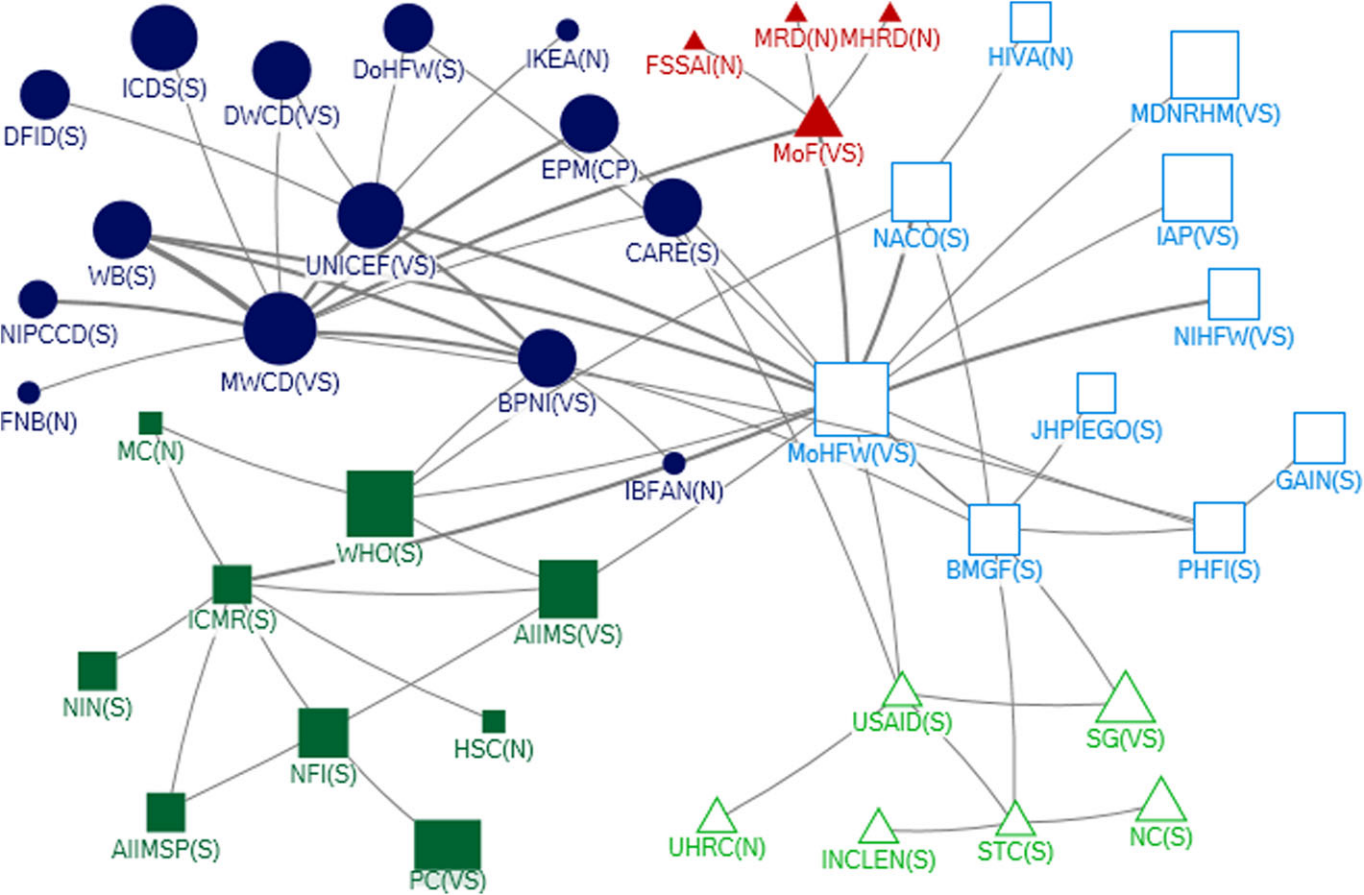
Community Network for Funding Support at National Level

We observed five communities within the funding network at National level (Fig. 3). The two largest communities were the MoWCD and MoHFW with the Ministry of Finance at the centre of a smaller community.

#### State level

**Fig. 4:**
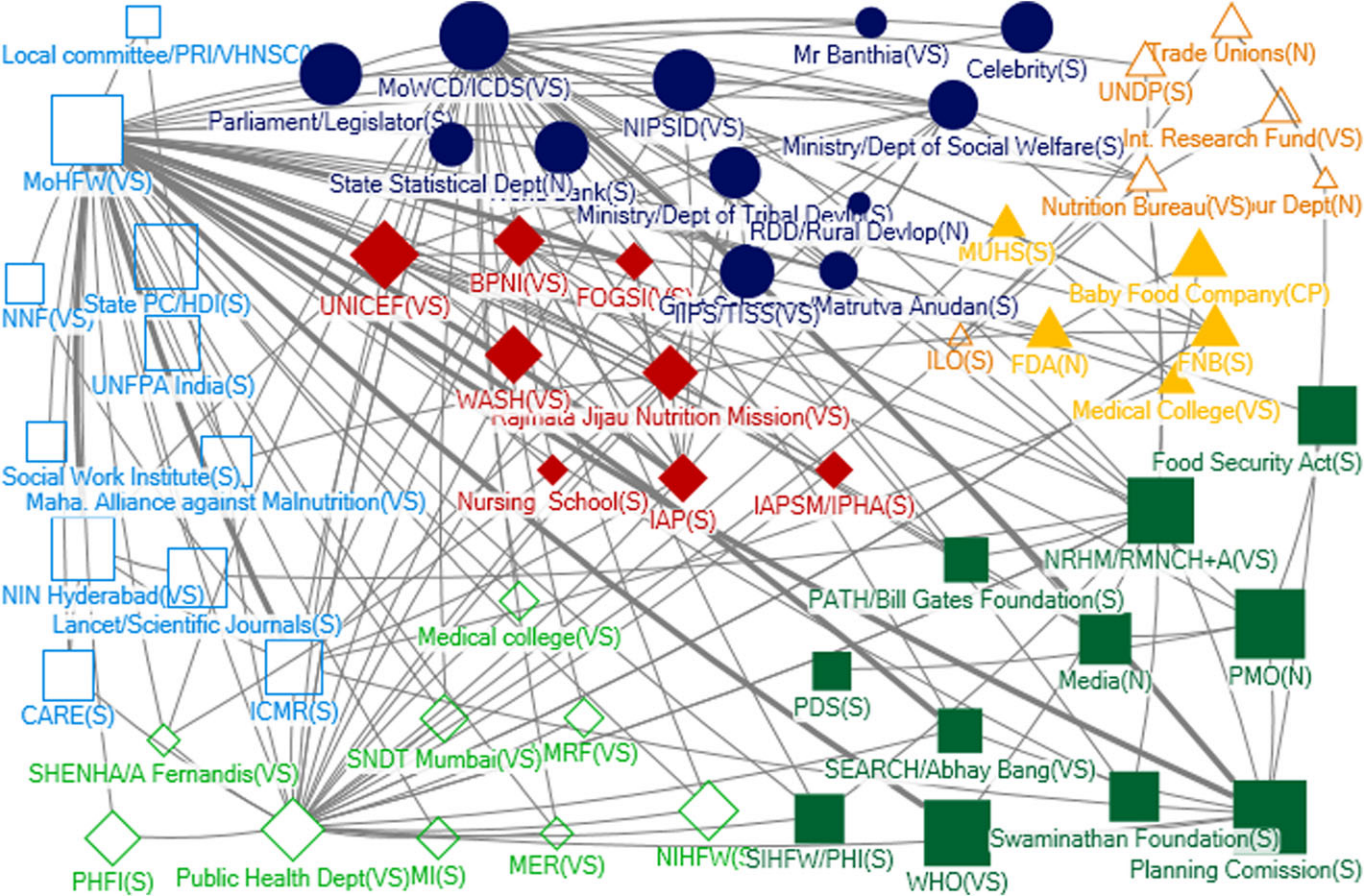
Community Network for Technical Support at Maharashtra State Level

**Fig. 5:**
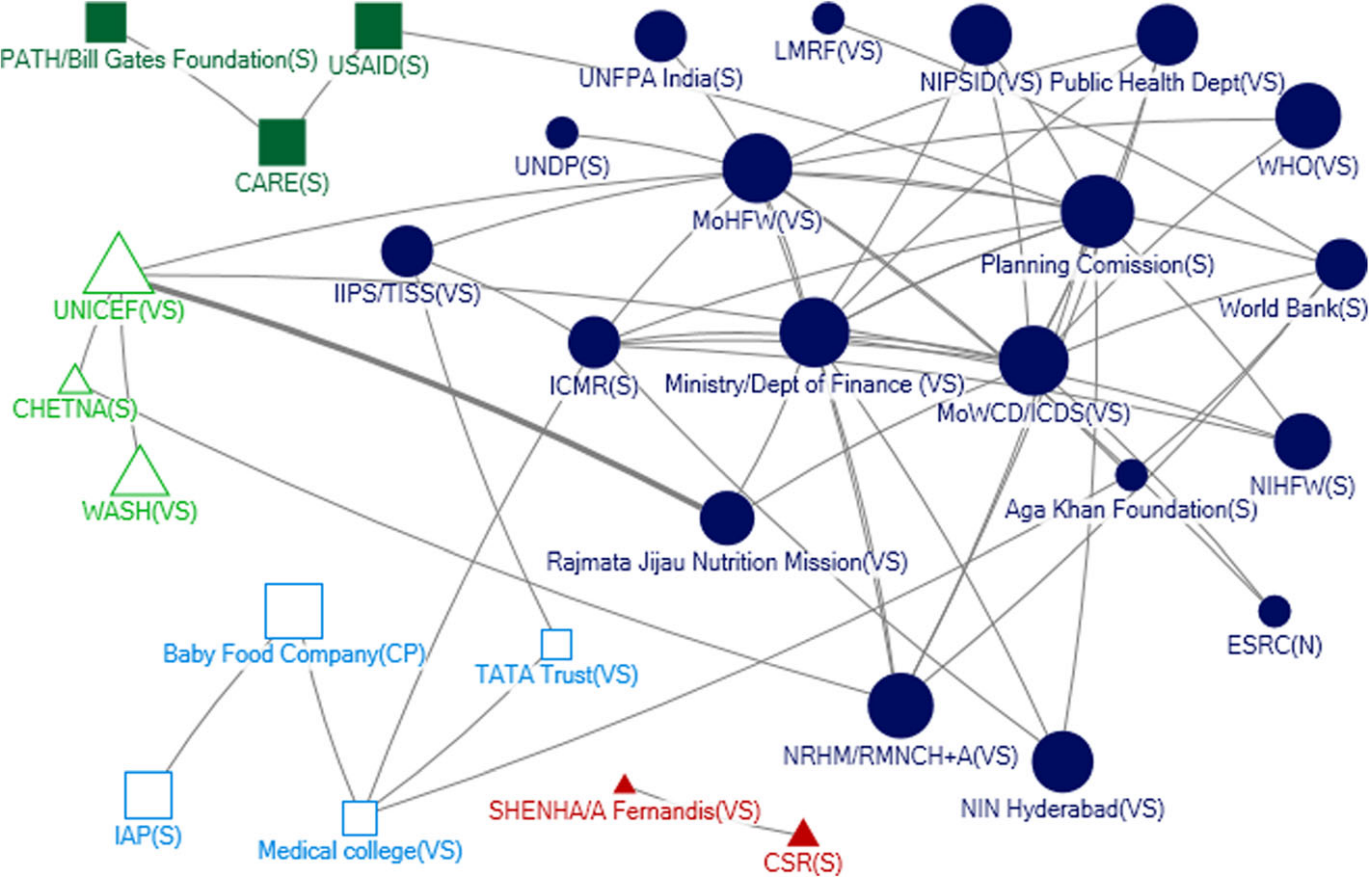
Community Network for Funding Support at Maharashtra State Level

**Fig. 6:**
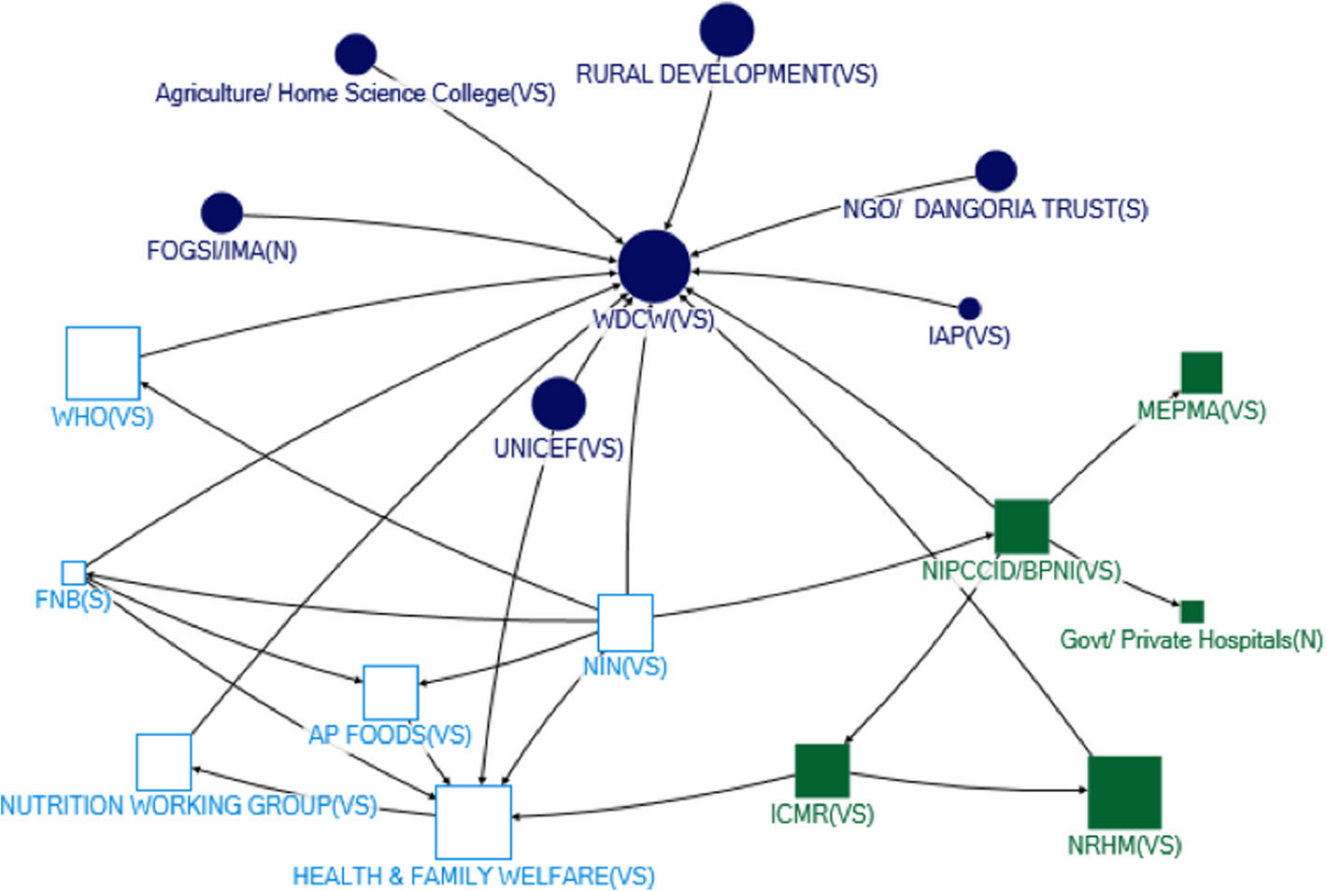
Community Network for Technical Support at Andhra Pradesh (Unified) State Level

**Fig. 7:**
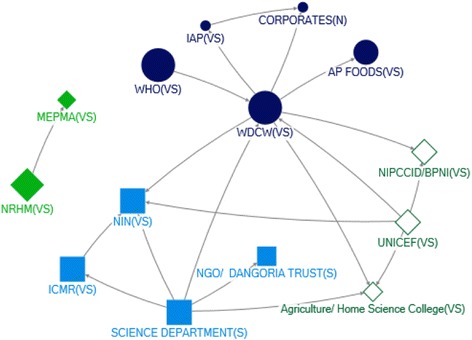
Community Network for Funding Support at Andhra Pradesh (Unified) State Level

Many actors identified as influential for IYCF, through technical or funding support, also appeared on the state level policy landscape - in particular, MoHFW and MoWCD (Table 2, Figs. 4, 5, 6 and 7). In Net-map discussion, respondents stated that MoHFW and MoWCD are the two key actors in IYCF policy and implementation. In addition, in both states, the Public Health Department (PHD) received technical support (in-degree), was accessible to a wide range of actors (closeness), and exerted a high degree of control over the flow of technical support (betweenness). This was evident from the net-map group discussion. One respondent stated

*“…… Public Health Department, comes under MoHFW, is mostly responsible for implementation of various IYCF related activities at district level and PHD is supported by ICDS, which comes under MoWCD, in implementation of IYCF program.”*

Rajmata Jijau Nutrition Mission, the Maharashtra state specific initiative, was also among the top five actors in terms of receiving technical support in that state (in-degree). Respondents stated that Rajmata Jijau Nutrition Mission is a high-priority initiative in Maharashtra state addressing the issue of child malnutrition through nutrition interventions, of which IYCF is one component. One respondent stated

*“…nutrition mission has made a significant contribution at state level in recommending innovative guidelines to promote and support IYCF…. Hirkani Kaksh (Baby care/ Feeding Room) at public Bus Stop is one of them”.*

One major difference was the prominence of NGOs in the state-level maps. Influential sources of technical support at state level were UNICEF and the Indian Council of Medical Research (ICMR). In Maharashtra, BPNI, IAP and the MoWCD were also in the top five. UNICEF, BPNI, IAP and ICMR provide technical support to IYCF primarily in formulation of operational guidelines and in training service providers. In Andhra Pradesh, NIN (based in Hyderabad) was one of the top five influential actors as providers of technical support (in-degree), accessibility in the network (closeness) and being strategically positioned (betweenness) – although most actors in the top five with regards to all four network measures were from the government sector (Table 2).

We observed seven communities in Maharashtra and four in Unified AP in the technical support network (Figs. 4, 5, 6 and 7). In Maharashtra, communities were heavily interlinked with separate communities forming around different influential government agencies (in particular, MoHFW, MoWCD, the state nutrition mission, and the PHD). In Unified AP, the communities were more diverse.

The Ministry of Finance and the Planning Commission were influential in both states as providers of funding (out-degree), and were strategically positioned in both networks (closeness and betweenness). The MoHFW was also a strategically positioned receiver of funding in both states. In Unified AP, the MoWCD and NRHM were both in the top five for all network measures – they were influential as providers and receivers of funding, and strategically positioned. Influence through funding was more diverse in Maharashtra, with influential actors from the government sector including the PHD (receiving funding) and MoWCD (receiving funding and strategically positioned), international agencies including the World Bank and UNICEF (providers of funding), and local non-government agencies such as the ICMR (strategically positioned provider of funds) (Table 2). This may result because the Planning Commission in the National Strategic Plan allocates budgets for programs, which are disbursed by the Ministry of Finance. Most activities for IYCF are implemented by MoHFW through PHD, hence MoHFW may be receiving major funding support for IYCF in addition to MoWCD.

We examined the funding network in five communities in each state (Figs. 6 and 7). In unified AP, all communities contained one or two large international funders (e.g. UNDP, UNICEF, World Bank) as well as government actors. In contrast, the funding network in Maharashtra state was characterized by one much larger community with a diversity of actors – including most of the government actors, indicting a high level of integration – and largely non-government agencies and other international funders in the other four small communities. One concern was the integration of corporate funders into some communities in both states.

In both states, Federation of Obstetrics and Gynaecological Societies of India (FOGSI) is almost missing in the IYCF policy scenario. However qualitative data showed that many stakeholders felt FOGSI may be a strategic partner for promoting and supporting IYCF by providing technical support to frame guidelines as well as implementation of activities because IYCF messages can be delivered during antenatal visits. Respondents felt pregnancy is a good opportunity to promote early initiation and EBF since the receptivity of messages would be high and would help prepare mothers for the period post birth. It was also felt that lactation cells, breast feeding support groups or a help line would be beneficial with more nuclear families and many young mothers not well educated in breastfeeding issues.

The network analysis followed in this manuscript identified the followings: influential actors in respect of different network measures; current patterns of actors working in small groups or communities etc. – these all lead to effective design of future policies for IYCF.

## Conclusions

The Indian government has made significant advances in policy and guidelines in child health and nutrition such as promulgating the National Food Security Act, assurance of maternity protection and food security for children, and restructuring ICDS, which now has more comprehensive provisions related to IYCF. Although there are legislative provisions to promote access to maternity leave and workplace support for women working in informal or unorganized sectors, these could be improved by including women in non-government and informal employment. One key strength is the integration of IYCF into a range of policy agendas and guidelines related to child health and development at both national and state levels. However, the lack of a specific national policy on IYCF means there is no formal mechanism for reviewing and monitoring of implementation across sectors and jurisdictions. A national implementation action plan would enable a stronger commitment towards IYCF and more effective application of IYCF programs.

Opportunities for strengthening policies would be cross-referencing policies or linking policies across various sectors such as MoHFW [RCH/NRHM], MoWCD (ICDS), legislation/law [IMS Act and maternity benefit]. India also needs clear planning and policy guidelines regarding IYCF in emergencies and tribal populations and legislations need framework for easier and rapid reporting of violations and to enable community monitoring.

Overall, the net-map analysis identified the most influential actors in the IYCF policy landscape for technical as well as funding support. This information may be useful for targeting advocacy efforts at state and national levels to generate a conducive policy environment for supporting, promoting and protecting IYCF in India.

## Additional file

Additional file 1: Table S1. Matrix showing domains addressed by IYCF and related policies. (DOCX 41 kb)
