# Supplementary material for: Policy content and stakeholder network analysis for infant and young child feeding in India
Source: BMC Public Health. 2017 Jun 13;17(Suppl 2):461. doi: 10.1186/s12889-017-4339-z (PMC5496016; doi:10.1186/s12889-017-4339-z)
Supplement: Additional file 1: Table S1. — Matrix showing domains addressed by IYCF and related policies. (DOCX 41 kb) [file 12889_2017_4339_MOESM1_ESM.docx]

| **Name of Policy** | **Policy - source** | **Support for IYCF** | | | | | | | **Policy description** |  | |  |
| --- | --- | --- | --- | --- | --- | --- | --- | --- | --- | --- | --- | --- |
|  |  | General support for infant and young child feeding | | Provision of correct information to mothers / caregivers | Training of health workers to counsel mothers | | Enable mothers to engage with best practice interventions | |  |  | |  |
| **Government Strategic Plans and Legislations** | | | | | | | | | | |  | |
| 12^th^ Five Year Plan (2012-2017) | Planning Commission 2012 | | X | X | X | | x | | - Implementation of IMS Act Amended 2003 - regulate IMS code, stopping of direct advertisement, protect promote and support IYCF - Nutrition Resource Platform - Nutrition and Education and Social Mobilization |  | |  |
| 5 year strategic plan Ministry of Women Children Development (2011-2016) | Ministry of Women & Child Development | | X | X | X | | X | | - Enhance budgetary support for Exclusive Breast Feeding and Complimentary Feeding. - Strengthening ICDS and NRHM. - Regulation of IMS code - Support for pregnant and lactating women through the conditional Maternity Benefit Scheme – Indira Gandhi MatritvaSahayogYojna. - Implementing the Rajiv Gandhi Crèche Scheme for Children for Working Mothers |  | |  |
| Indian National Code for Protection and Promotion of Breast-feeding (Act of Parliament) | Ministry of Social Welfare, 1983  (Resolution No. 18-11/81-NT] | | X | X |  | | X | | - Correct marketing practices - Controls manufacturers of breast milk substitutes - Planning, provision , design and dissemination of information - Implementation through legislation and other appropriate measures. |  | |  |
| The Infant Milk Substitutes, Feeding Bottles and Infant Foods (Regulation of Production, Supply and Distribution Act, 1992 Amended in 2003 (IMS Act) | Department of Women & Child Development; Ministry of Human Resource Development  Legal Act passed by Parliament | | X | X |  | | X | | - Legal framework for regulatory approach to promote breast feeding of new born children and infants. - Regulation of the production, supply and distribution of infant milk substitutes, feeding bottles and infant foods for the protection and promotion of breastfeeding and ensuring proper use of infant foods. |  | |  |
| The Maternity Benefit Act - 1961  Amended in 2007 | Ministry of Labour & Employment | | X |  |  | | X | | - Maternity benefit - 6 months but has no mention of IYCF or related counseling. |  | |  |
| Central Civil Services Leave Rules; 1972 | Ministry of Personnel and Public Grievances and Pensions | |  |  |  | |  | | - Maternity and paternity leave benefits but has no mention of IYCF or related counseling. |  | |  |
| The Employees’ State Insurance Act, 1948 |  | |  |  |  | |  | | - Maternity benefits for insured persons. |  | |  |
| ***National Policies and Plans of Action*** | | | | | | | | | | |  | |
| National Nutrition Policy, 1993 | Department of Women & Child Development  Ministry of Human Resource Development | |  | X | |  | | X | - Popularize low cost nutritious food including weaning foods , - Dissemination of basic health and nutrition knowledge with special focus on wholesome infant feeding practices - Growth monitoring |  | |  |
| National Plan of Action on Nutrition 1995 | Department of Human Resource Development  Ministry of Human Resource Development | | X |  | | X | | X | - Ensuring that the information disseminated on the feeding of infants and young children is consistent and in line with current scientific knowledge and provisions of the Infant Milk Substitutes Act 1993 - Empowering all mothers to breast feed their children exclusively for first four to six months and to continue breast feeding with complementary food well into the second year - Ensuring effective collaboration with infrastructure of Integrated Child Development Services (ICDS), Food and Nutrition Board (FNB), |  | |  |
| National Policy for Children, 2012 | Ministry of Women & Child Development | | X | X | |  | | X | - Support to women for exclusive breastfeeding through universal provision of maternity entitlements and universal access to crèche facilities. - Right to Adequate Nutrition - HIV & nutrition |  | |  |
| The National Plan of Action for Children, 2005 | Department of Women & Child Development  Ministry of Human Resource Development | | X |  | | X | |  | - ensure maternity entitlements to all women in order to reduce unsafe births and neonatal, Infant & Maternal Mortality and encourage breast feeding - ensure crèches at working places so that all young children of working women receive essential care and protection while women work - evolve a National Program to empower families for new born care, temperature management, breast-feeding and identifying warning signs and provide essential newborn care through skill building in community level workers - ensure the feeding of new-borns with colostrum (first breast milk) in both home and institutional deliveries. |  | |  |
| National Health Policy 1983 revised in 2002 | Ministry of Health and Family Welfare Government of India | | X |  | |  | |  | - Give special benefits to women & child through public health system |  | |  |
| National Population Policy, 2000 | Ministry of Health and Family Welfare Government of India | | X | X | |  | | X | - To promote the reproductive and child health care through mobile clinics and counselling services of Health - The Baby Friendly Hospital initiatives (BFHI) should be extended to all hospitals and clinics. - Crèches and child care centres will be opened in rural areas and urban slums but do not mention IYCF |  | |  |
| ***Guidelines/ Implementation Documents*** | | | | | | | | | | |  | |
| Guidelines for Enhancing Optimal Infant and Young Child Feeding Practices, 2013 | Ministry of Health and Family Welfare Government of India | | X | X | | X | | X | - Health System strengthening and support, from National level to Village level - Planning of National Apex body for IYCF – Child Health Division, MoHFW - National Training Centres for IYCF - National Technical Nutrition Support Unit - State Coordination Group and Resource Centres for Nutrition (SCRCN) - Developing a detailed Nutrition Action Plan for State and Districts - State and District Level Awareness generation for the IMS Act |  | |  |
| Dietary Guidelines for Indians, 2010 | Ministry of Health and Family Welfare Government of India | | X | X | |  | | X | - Promote exclusive breastfeeding for six months and encourage breastfeeding till two years. |  | |  |
| National Guidelines on Infant and Young Child Feeding 2003 Revised 2006. | Department of Women Child Development, Ministry of Health Research Development | | X | X | | X | | X | - Media and Voluntary Organization effective implementation of these guidelines. |  | |  |
| Operational Guidelines Nutrition Rehabilitation Centre (NRC)  Operational Guidelines on Facility Based Management of Children with Severe Acute Malnutrition, 2011 | Ministry of Health and Family Welfare Government of India | | X | X | | X | | X | - IEC Campaigns - Focused BCC training modules |  | |  |
| Integrated Management of Neonatal and Childhood Illness (IMNCI) 2009 | Ministry of Health & Family Welfare, Government of India | | X | X | | X | | X |  |  | |  |
| Guidelines for HIV Care and Treatment in Infants and Children, 2006: | Ministry of Health & Family Welfare, Government of India | | X | X | | X | | X | - Guidelines for care of HIV exposed infants. |  | |  |
| Integrated Child Development Services Scheme | Ministry of Women & Child Development | | X | X | | X | | X | - Food Nutrition Board will integrate IYCF into ICDS, NRHM |  | |  |
| JananiSurakshaYojna (National Rural Health Mission) | Ministry of Health and Family Welfare Government of India | | X |  | |  | |  | - Cash Transfer to contribute to food and nutrition security for 0-6 months infants |  | |  |
| Rajiv Gandhi National Crèche Scheme for the Children of Working Mothers | Government of India | | X |  | |  | |  | - Mothers’ breastfeeding their babies can conveniently come to feed their babies. - Supplementary nutrition register for recording the food provided to the children |  | |  |
| Indira Gandhi MatritvaSahayogYojna. | Ministry of Women and Child Health | | X |  | |  | |  | - Cash Incentives for improved health and nutrition to lactation mothers - Effective convergence with health department |  | |  |
| JananiShishuSurakshaKarykram | National Health Mission | |  |  | |  | |  | - Entitlements and elimination of out-of-pocket expenses for both pregnant women and sick neonates |  | |  |
| NAVJAAT SHISHU SURAKSHA KARYAKRAM (Basic newborn care and resuscitation program training manual) | Ministry of Health and Family Welfare Government of India | | X |  | | X | |  | - Encourage the initiation of breastfeeding. - Demonstrate various positions for breastfeeding a baby - Assessing the adequacy of breastfeeding by health personnel |  | |  |
| ***Maharashtra*** | | | | | | | | | | |  | |
| RajmataJijau Health and Nutrition campaign | Ministry of Women and Child Development 2012 | | X |  | |  | |  | - At working place established “Hirkanikaksh”(Hirakani room) for feeding baby - Established Milk Bank for employed women - Give Nutrition Demonstrations to women |  | |  |
| ***United Andhra Pradesh*** | | | | | | | | | | |  | |
| Anna AmruthaHastham (AAH) | Department Women Development and Child Welfare-Jan 2013 | | X | X | |  | |  | - The scheme envisages provision of “One full meal” to pregnant and lactating women daily. It is intended to provide nutritious food to the beneficiaries and thereby reduce the incidence of infant and maternal mortality rates. The nutritional intervention will help reduce anaemia among pregnant women and prevent low birth weight infants |  | |  |
| Balamurtham | Department Women Development and Child Welfare 2014 | | X | X | | X | |  | - Balamurtham is the weaning food introduced under ICDS to provide improved supplementary nutrition to children between 7 months and 3 years. The food is a preparation of wheat, channa dhal, milk powder, oil and sugar. It is fortified and provides 50% of RDA. - Along with counselling on IYCF practices Balamutham is envisaged to reduce malnutrition |  | |  |
| Special Care and Supervised feeding of malnourished children (0-6) | Department Women Development  and Child Welfare- 2014 | | X | X | |  | |  | - Guidelines have been issued for identifying malnourished children up to 5 years by weight for age criteria, by weight for height criteria and by presentation of bilateral pitting oedema and to take. Special care and Supervised feeding of such children. |  | |  |
| Convergence to improve Health and Nutrition Status of Women and Children - Interdepartmental Coordination for Effective Convergence –Maarpu Programme | Health, Medical & Family Welfare Department-  2013 | | X | X | | X | | X | - Focus on 20 key interventions to reduce MMR, IMR & Malnutrition. II. Convergence in Service Delivery at the habitation level. III. Convergent Behavioural Change Communication (BCC). IV. Monitoring of the 20 key interventions. V. Participation of SHGs & Village Organisations (VOs) VI. Use of Maternal and Child Protection (MCP) card. VII. Synchronization. VIII. Administrative Structures for convergence. - Nutrition and Health Days are conducted twice a month |  | |  |
